# Personalized Medicine: Pharmacokinetics

**DOI:** 10.3390/jpm12101660

**Published:** 2022-10-06

**Authors:** Youssef Daali

**Affiliations:** Division of Clinical Pharmacology and Toxicology, Geneva University Hospitals, 1205 Geneva, Switzerland; youssef.daali@hcuge.ch; Tel.: +41-223795430

The “one-size fits all” model that has been used for decades is now replaced by the concept of the “right dose of the right drug for the right patient”. However, its use in clinical setting is not yet a common practice, due to many factors, such as healthcare professionals’ education, absence of large clinical validation studies, and the complexity and interplay between sources of variability [1]. Adverse drug reactions (ADRs) are responsible of more than 6% of all hospitalizations and represent the fourth most common cause of death in developed countries [2]. The consequences of ADRs are a decrease in health condition and in quality of life, an increase in heath cost and, for the pharmaceutical industry, a risk of withdrawal from the market with colossal economic costs. Interindividual variability is due to genetic and environmental factors affecting pharmacodynamics as well as pharmacokinetics. While pharmacodynamic factors affecting drug responses are not well characterized, pharmacokinetic sources of variability have been deeply studied and established. Genetic and environmental factors such as drug–drug interactions, organ failure, inflammation and disease are responsible for pharmacokinetics variability. Identification and clinical validation of these factors in large cohorts is crucial if we are to enter the era of precision medicine. Metabolic enzymes (phase I and phase II) and transporters play major roles in variability. Cytochromes P450 (CYPs) are the most important metabolic enzymes responsible of the clearance of drugs. Genetic polymorphisms affecting the activity of CYPs are well described in different knowledge bases such as PharmGKB; different organizations such as the Clinical Pharmacogenetics Implementation Consortium (CPIC) and the Dutch Pharmacogenetics Working Group (DPWG) edit guidelines based on genetics for tailored medication [3]. However, genetic polymorphisms alone, without considering environmental factors, explain only part of the variability. Drug–drug interactions, food, herbs, inflammation and disease can considerably affect the pharmacokinetics of drugs. Recently, transporters became an important player in pharmacokinetics. These proteins are also subject to genetic polymorphisms and many drugs are substrates or inhibitors. For example, statins are known to be substrates of the organic anion transporter polypeptide OATP1B1; genetic polymorphisms and/or inhibition of this protein are responsible of ADRs such as myopathy, rhabdomyolysis, elevated liver enzymes and acute kidney injury [4]. Anticancer platinum agents’ nephrotoxicity was reported to occur because of their interaction with organic cation transporter OCT/SLC22A, and multidrug and toxin extrusion MATE/SLC47A [5].

Genotyping and phenotyping of metabolic enzymes and transporters, in combination with the use of model-informed precision dosing approaches, such as population pharmacokinetics and physiologically based pharmacokinetics (PBPK), can be used in clinical settings for optimal therapy.

This Special Issue is dedicated to the role of pharmacokinetics in precision medicine. Dujic et al. reported a drug–drug interaction between omeprazole and Gliclazide via CYP2C19 inhibition. Authors used PBPK to predict the extent of interaction and found that omeprazole increases exposure to gliclazide with elevated risk of gliclazide-associated hypoglycemia. Fernandez et al. published a systemic review on drug–drug interactions leading to ADRs with the anticoagulant rivaroxaban. Pharmacokinetic interactions were mostly with CYP3A/P-gp modulators and the majority of interactions were in pharmacodynamics. Magliocco et al. proposed a new endogenous marker of the activity of CYP3A by measuring the 1β-hydroxy-deoxycholic acid (1β-OH-DCA)-to-deoxycholic acid (DCA) urinary metabolic ratio. 1β-OH-DCA/DCA correlated significantly with oral MDZ clearance and the modulation of CYP3A was reflected in the 1β-OH-DCA/DCA UMR after the intake of rifampicin (induction ratio = 11.4, *p* < 0.01). Rollason et al. evaluated the link between an ADR or a non-response to treatment and CYPs, P-glycoprotein (P-gp) or catechol-O-methyltransferase (COMT) activity in patients taking analgesic drugs for chronic pain. Authors showed that the genotypic and phenotypic approach is useful to understand ADRs or therapeutic resistance and can be part of the evaluation of chronic pain patients. Jantararoungtong et al. investigated the frequency of Thiopurine methyltransferase variants (TPMT 719A > G (*3C), ITPA 94C > A and ITPA 123G > A) and drug transporter variants (MRP4 912 C > A and MRP4 2269G > A) in Thai children with ALL and their association with 6-MP-related adverse events. They confirmed the association between TPMT*3C and 6-MP-induced myelotoxicity. Waespe et al. published a review describing the genetic predictors for sinusoidal obstruction syndrome (SOS). Evidence for a significant association of genotypes with SOS was found for GSTA1 variants. Two papers related to pharmacodynamic targets were published in this Special Issue: the first, by Elhourch et al., showed the association between polymorphisms in the TCF7L2 gene with a higher risk of type 2 diabetes in a Moroccan population; the second, by Garcia et al., identified circulating miRNAs associated with platelet aggregation and platelet-supported thrombin generation.

We would like to thank all authors for their participation in the success of this Special Issue and the quality of submitted papers.

## References

[1] Storelli F., Samer C., Reny J.L., Desmeules J., Daali Y. (2018). Complex Drug-Drug-Gene-Disease Interactions Involving Cytochromes P450: Systematic Review of Published Case Reports and Clinical Perspectives. Clin. Pharmacokinet..

[2] Cacabelos R., Cacabelos N., Carril J.C. (2019). The role of pharmacogenomics in adverse drug reactions. Expert Rev. Clin. Pharmacol..

[3] Volpi S., Bult C.J., Chisholm R.L., Deverka P.A., Ginsburg G.S., Jacob H.J., Kasapi M., McLeod H.L., Roden D.M., Williams M.S. (2018). Research Directions in the Clinical Implementation of Pharmacogenomics: An Overview of US Programs and Projects. Clin. Pharmacol. Ther..

[4] Balasubramanian R., Maideen N.M.P. (2021). HMG-CoA Reductase Inhibitors (Statins) and their Drug Interactions Involving CYP Enzymes, P-glycoprotein and OATP Transporters-An Overview. Curr. Drug Metab..

[5] Yonezawa A., Inui K. (2011). Organic cation transporter OCT/SLC22A and H(+)/organic cation antiporter MATE/SLC47A are key molecules for nephrotoxicity of platinum agents. Biochem. Pharmacol..

